# Psychopathy, adaptation, and disorder

**DOI:** 10.3389/fpsyg.2013.00139

**Published:** 2013-03-27

**Authors:** Daniel Brian Krupp, Lindsay A. Sewall, Martin L. Lalumière, Craig Sheriff, Grant T. Harris

**Affiliations:** ^1^Department of Mathematics and Statistics, Queen's UniversityKingston, ON, Canada; ^2^Department of Psychology, Queen's UniversityKingston, ON, Canada; ^3^Department of Psychology, University of SaskatchewanSaskatoon, SK, Canada; ^4^School of Psychology, University of OttawaOttawa, ON, Canada; ^5^Tulloch Mapping SolutionsOttawa, ON, Canada; ^6^Waypoint Centre for Mental HealthPenetanguishene, ON, Canada

**Keywords:** psychopathy, mental disorder, adaptation, social evolution, inclusive fitness theory

## Abstract

In a recent study, we found a negative association between psychopathy and violence against genetic relatives. We interpreted this result as a form of nepotism and argued that it failed to support the hypothesis that psychopathy is a mental disorder, suggesting instead that it supports the hypothesis that psychopathy is an evolved life history strategy. This interpretation and subsequent arguments have been challenged in a number of ways. Here, we identify several misunderstandings regarding the *harmful dysfunction* definition of mental disorder as it applies to psychopathy and regarding the meaning of nepotism. Furthermore, we examine the evidence provided by our critics that psychopathy is associated with other disorders, and we offer a comment on their alternative model of psychopathy. We conclude that there remains little evidence that psychopathy is the product of dysfunctional mechanisms.

Psychopathy poses a significant problem to communal living: psychopaths are far more likely than others to engage in harmful behaviors ranging from the parasitic to the homicidal. In this light, we thank Leedom and Hartoonian Almas (2012) for their comments on Krupp et al. (2012), as they provide us with an opportunity to clarify the arguments we made and to continue working toward a sophisticated and testable evolution-minded model of psychopathy that may help to address this problem.

Following Wakefield's (1992) influential concept of *harmful dysfunction*, Krupp et al. (2012; see also Harris et al., 2001a,b) argued that psychopathy may be considered a mental disorder only if there is compelling evidence that it (1) leads to harm to self or others *and* (2) is caused by a failure of psychological mechanisms to serve the functions for which they were designed by natural selection. In keeping with the first criterion, we accept without qualification that psychopathy causes disproportionate harm to others. What we challenge is the claim that there is evidence, compelling or otherwise, of the second criterion—that psychopathy is the result of dysfunctional psychological mechanisms in the sense implied above.

We take it as given that the brains of psychopaths differ from those of nonpsychopaths in systematic ways. Without such differences, psychopaths could not be reliably set apart in their cognition and behavior from nonpsychopaths. But *difference* is not isomorphic with *dysfunction*. For instance, although the brains of men and women have much in common, they must also be different on average, as must the brains of young and old, married and single, androphile and gynephile, Anglophone and Francophone, and so on, even if these brain differences are solely the result of differences of experience. While the life sciences have begun to recognize that such differences do not inherently reflect disorder, the relationship between difference and disorder nevertheless continues to bedevil the study of mental health.

An argument for dysfunction must marshal supporting evidence, and this must be distinguishable from evidence of difference. For example, researchers should not assume without corroboration that a burglar was mentally disordered simply because he committed theft, any more than they should assume that a child was mentally disordered for failing to share her toys with her siblings. *Chronic* malefaction, however, is often construed—groundlessly, we hasten to add—as proof of disorder. And so, while researchers enthusiastically catalogue the neural and cognitive particulars of inveterate misbehavior, their efforts do not necessarily provide evidence of the failure of mechanisms to perform their selected functions.

There are many signs that mental mechanisms are not performing in accordance with their adaptive design, including injuries to the brain, obstetrical complications, excessive levels of fluctuating asymmetry, and other indications of neurodevelopmental perturbation. As discussed in our article and the references cited therein, there is no evidence that any of these indicators covary positively with psychopathy. Other signs of mental dysfunction might include behaviors that would have led to ineluctable inclusive fitness costs in ancestral environments. An individual's inclusive fitness is the net effect of its genotype on its own fitness (*d*, for direct fitness), including effects on lineal descendants, and on the fitness of others bearing nonrandom genotypes (*i*, for indirect fitness), including effects on collateral kin (Hamilton, 1964, 1970). Upregulating, downregulating, or taking adaptations entirely “offline” may have led to inclusive fitness decrements or increments, depending upon the conditions under which these adaptations would have been expected to operate. For instance, worker sterility has independently evolved numerous times among the eusocial Hymenoptera and other eusocial taxa (e.g., aphids, termites, and thrips). What might appear at first blush to be an unmitigated failure of design—the reproductive function of scores of individuals has been downregulated or taken offline, in many cases permanently—is anything but: eusociality has evolved only under conditions of high genetic relatedness between reproductives and workers, in keeping with the hypothesis that workers benefit their inclusive fitness more from rearing their full siblings together than from rearing their own offspring alone (Hughes et al., 2008). Hence, even gross variation in behavioral or physical phenotype may be due either to developmental error and injury or to adaptive design, in much the same way that the polymorphisms of the army ant *Eciton burchellii* (Franks, 1985, 1986) or male bluegill sunfish (*Lepomis macrocheris*; Gross and Charnov, 1980; Gross, 1991) reflect adaptive specialization to different strategic interests.

The task at hand, then, is to discover whether differences in mental functioning historically entailed inclusive fitness costs and to furthermore identify their sources. For example, failing memory systems cause profound deficits in the ability to navigate one's physical and social universe, as evidenced by individuals with anterograde amnesia (Scoville and Milner, 1957), dementia (Bowen et al., 1997), and schizophrenia (Frith and Done, 1989; Nathaniel-James and Frith, 1996; Corcoran and Frith, 2003). These memory failures seem neither to provide any fitness advantage nor to be compensated for by modified or alternative mechanisms, and so would have caused a decrease in the inclusive fitness of those afflicted with them. Concomitant with these failures are indicators of pathology and disturbance, including brain damage (Scoville and Milner, 1957), minor physical anomalies (Weinberg et al., 2007), pre- and perinatal complications (Cannon et al., 2002), excess fluctuating asymmetry (Markow, 1992), and oxidative stress (Bennett et al., 2009; Bitanihirwe and Woo, 2011), as would be expected were they symptomatic of a mental disorder.

The inclusive fitness effect is the maximand of selection (Grafen, 2006), and the corresponding condition for which an action is favored by selection is given by *d* + *i* > 0. Using this formulation, it becomes plain that individuals pursuing a psychopathic “strategy” as we and others have envisioned it—one of persistent social exploitation—must, on balance, behave in ways that historically maximized benefits and minimized costs to *d, i*, or both. Thus, for a start, they cannot impose costs on their lineal descendants and on collateral kin that, after weighting by relatedness, are disproportionate to the benefits of their actions. Psychopathy seems to be associated with an upregulation of sexual psychology and associated behaviors (Quinsey et al., 1995; Lalumière and Quinsey, 1996; Harris et al., 2007b), and this is mirrored by evidence that psychopathic offenders have at least as many offspring as nonpsychopathic controls and may even have slightly more (Harris et al., 2007b; Pulkkinen et al., 2009; Vachon et al., 2012; cf. Power et al., 2013). We do not know with any certainty what fecundity advantage psychopaths would have enjoyed in ancestral environments, but its effects on inclusive fitness would have been undercut by any imposition of significant costs to offspring and other genealogical kin.

Following the logic outlined above, the arguments for dysfunction and for adaptation generate rival hypotheses. If, on the one hand, psychopathy is a mental disorder *sensu* Wakefield (1992), then we would hypothesize that psychopathic offenders, relative to their nonpsychopathic counterparts, ought not to behave nepotistically, because their psychology has been profoundly affected by a maladaptive course of development. After all, it would be peculiar for misdirected ontogenetic processes to leave nepotistic devices alone when they have had such sweeping, downregulatory effects on related systems, such as empathy, remorse, and guilt (Hare, 1993). Indeed, as discussed in Krupp et al. (2012), perpetrators who behave violently toward kin carry an elevated risk of mental disorder diagnosis (Daly and Wilson, 1988a; Harris et al., 2007a). If, on the other hand, psychopathy represents an alternative life strategy of persistent social exploitation, then we would hypothesize that psychopaths ought to behave nepotistically in that they limit the costs they impose on genetic relatives. Thus, nepotistic discrimination can be used as an indicator of mental disorder.

In our study, we failed to support the former hypothesis and, consequently, supported the latter: scores on the Psychopathy Checklist-Revised (PCL-R; Hare, 1991), an archetypal measure of psychopathy, were significantly negatively associated with victim-offender relatedness in the index offenses of violent offenders. This finding, which implies that psychopathy is associated with benefits rather than costs to inclusive fitness (because harm was likely allocated to negative relatives), was not explained by measures of distance or frequency of dispersal, and it was not explained by a measure of inbreeding avoidance, though it must of course be explained by *something*. When the sample was restricted to offenders who did not coreside with kin, the direction of the association remained unchanged, though it was no longer statistically significant, continuing to imply that psychopathy was not associated with any cost to an individual's inclusive fitness. All of this suggests that, in the context of violence, psychopathic offenders behaved at least as nepotistically, and possibly more so, than did their nonpsychopathic counterparts.

Broadly, Leedom and Hartoonian Almas (2012) criticize Krupp et al. (2012) from two positions. Their first point of disagreement regards evidence of nepotism among psychopathic individuals. While they do not dispute our findings, they argue that these findings do not constitute evidence of nepotism for three reasons: (1) psychopaths may nevertheless harm genealogical relatives in ways not measured by our study; (2) a majority of psychopathic offenders are nonviolent, and so our use of violence as a measure of harm is inappropriate; and (3) a reduction in harm to relatives is not the same as nepotistic help.

We acknowledged (1) when we wrote: “[i]t is possible, for instance, that the cumulative effects of psychopathy on an offender's kin, including negative reputational effects, are substantial, even though the costs of individual actions may be small” (Krupp et al., 2012, p. 5). Researchers are limited to the data they have in hand, however, and (as discussed by Leedom and Hartoonian Almas, 2012) no other study of sufficient quality exists that speaks to this problem. Regarding (2), violence would undoubtedly have had large effects on fitness relative to most other forms of social interaction, and so is relevant even when rare. Moreover, Leedom and Hartoonian Almas provide no evidence that “most psychopathic individuals are not violent” (p. 1). The PCL-R is a formidable individual-level predictor of violence in many forms (reviewed in Harris et al., 2001b); if we cannot apply to psychopathic individuals data measuring violent behavior, to whom can such data be applied? Finally, and furthermore with respect to (1), studies of violence may provide unique psychological insights, because violence is rarely caused by anything short of powerful concerns and because reports of violence are bound to be considerably less biased than reports of other sorts of behavior (Daly and Wilson, 1988a,b). To deny the importance of violence because it is rare is to deny all that studies of violence have contributed to our understanding of parental and familial love (e.g., Wilson et al., 1980; Daly and Wilson, 1982, 1988a,b; Harris et al., 2007a), sexual proprietariness (e.g., Daly et al., 1982; Daly and Wilson, 1988a,b; Wilson and Daly, 1992, 1993), and intrasexual competition (e.g., Wilson and Daly, 1985, 1997; Daly and Wilson, 1988a,b, 2010).

We are puzzled by (3), given that Leedom and Hartoonian Almas (2012) quote fully the following statement from Krupp et al. (2012, p. 2, emphasis added): “individuals executing well-designed strategies, a necessary feature of psychological adaptations, should tend to be nepotistic—providing aid to close genealogical kin *and/or sparing them from harm*.” As discussed here and in the original article, nepotistic systems are not required to help genealogical relatives; rather, they are required to discriminate between positive and negative relatives in such a way as to maximize the actor's inclusive fitness, which may entail helping kin when cooperation is favored *or* minimizing the costs to kin and instead imposing them on others when it is not. Simply put, a lack of violent harm need not be synonymous with help for it to qualify as an adaptive form of nepotism. Psychopathic individuals may not make for nice genetic relations, but they appear to make for worse nongenetic relations still.

Leedom and Hartoonian Almas's (2012) second point of disagreement concerns the way in which our study speaks to Wakefield's (1992) concept of mental disorder which, if the reader will recall, is defined as a psychological phenomenon that (1) causes harm to self or others and (2) is the result of a failure of one or more psychological mechanisms to perform the tasks for which they were designed. Curiously, Leedom and Hartoonian Almas focus only on the first criterion, stating that “Any ‘benefit’ of psychopathy according to Krupp et al. (2012) is in perpetuation of genes only. However, that psychopathic individuals might contribute to the gene pool, has no bearing on *the definition of harm* as conceptualized by Wakefield” (p. 1, emphasis added). This is true insofar as criterion (1) is concerned, but our analysis was concerned with criterion (2)—that the design of nepotistic psychology remains intact among psychopaths, providing further evidence in support of extant adaptation hypotheses and against mental disorder hypotheses. Leedom and Hartoonian Almas appear to have understood that all parties are in agreement on criterion (1), as they state “That psychopathy is associated with disastrous consequences for individuals and society was not disputed by the authors” (p. 1). Yet, they seem to have entirely overlooked the relevance of our study to criterion (2): the *only* measure of cost and benefit that matters with respect to Wakefield's (1992) dysfunction criterion is that which concerns the effect of an action on inclusive fitness.

Relatedly, Leedom and Hartoonian Almas (2012) disagree with our statement that “psychopathy is neither comorbid nor associated with the neurodevelopmental perturbations characteristic of other serious mental illnesses, such as psychosis and mental retardation” (Krupp et al., 2012, p. 1), arguing that psychopathy is associated with paranoid personality disorder (citing Blackburn and Maybury, 1985; Blackburn, 1998; Fullam and Dolan, 2006; McGregor et al., 2012) and Eysenck's concept of psychoticism (citing Eysenck and Eysenck, 1977; Corr, 2010); they further point to a study by Suchankova et al. (2012) showing that psychoticism is associated with schizophrenia. While we covered the issue of neurodevelopmental perturbations in Krupp et al. (2012) and elsewhere (Harris et al., 2001a; Lalumière et al., 2001), we must further examine the evidence that Leedom and Hartoonian Almas provide suggesting that psychopathy is comorbid with true mental illness. Unfortunately, we find this evidence wanting.

First, while psychopathy is comorbid with some personality disorders (e.g., antisocial personality disorder), none of them—paranoid personality disorder included—reflect psychosis. Moreover, it remains to be established whether any personality disorder may be characterized as a disorder in the sense meant by Wakefield (1992). Second, it is clear that Eysenck's concept of psychoticism is unusual, as it reflects tough-minded, antisocial, unempathic, egocentric, impulsive, and aggressive traits (Eysenck, 1992). These characteristics resemble psychopathy far more than they do psychosis. Third, neither Blackburn and Maybury (1985) nor Corr (2010) presented any data demonstrating that psychopathy and psychosis (as it is commonly understood) are comorbid; rather, they relied on associations between measures of psychopathy and Eysenck's psychoticism. All of this is tantamount to idiosyncratically defining psychosis as manifesting manipulation, callousness, lying, remorselessness, glibness, shallow affect, proneness to boredom, irresponsibility, impulsivity, poor behavior controls, criminal versatility (and so on), and then “discovering” that it is comorbid with psychopathy as assessed by the PCL-R. Fourth, Fullam and Dolan (2006) and McGregor et al. (2012) only studied patients with schizophrenia or schizophrenia spectrum conditions, reporting that PCL-R scores predicted violence in such samples; the title of Fullam and Dolan's article notwithstanding, this sort of work does not—indeed, cannot—bear on the question of whether psychopathy and schizophrenia are comorbid (i.e., associated with each other in the population). Finally, Suchankova et al. (2012) provided evidence of a link between schizophrenia and an amalgam of scores on the detachment and suspicion subscales of the Karolinska Scales of Personality, but it is not remotely clear how such a relationship speaks to the question of whether psychopathy and psychosis (as conventionally understood) are related.

Leedom and Hartoonian Almas (2012) argue that, “[r]ather than being ‘an adaptation’ psychopathy may represent a spandrel—a syndrome that arises as a consequence of other features” (p. 2). They then go on to sketch their own evolution-minded model of psychopathy as, if we understand it correctly, the upshot of selection on dominance systems. We cannot adequately address here whether this model better accounts for the unique features of psychopathy than do others. We can, however, point out that identifying psychopathy as a “spandrel” (Gould and Lewontin, 1979; see also Buss et al., 1998; Andrews et al., 2002) does not speak to the argument for dysfunction. A spandrel has no evolved function, and so cannot itself be dysfunctional. Hence, the mechanisms from which a spandrel manifests would themselves have to be dysfunctional to support an argument for dysfunction. In turn, the evidence for this would have to resemble that which has been outlined above: indications of neurodevelopmental perturbation and dysregulation of systems resulting in historical inclusive fitness costs. We arrive, then, back where we began: there is no evidence that psychopathy is the result of dysfunctional rather than merely different psychological mechanisms.

The disagreement between adaptation and disorder models of psychopathy does not rest solely on semantics. To the degree that one class of model better accounts for the psychology of psychopathy, it will also better help us to understand and, hopefully, to cope with psychopathy. If psychopathy is a mental disorder, the absence of indicators of developmental instability or intellectual, operational, or reproductive disadvantage strikes us as odd. Equally surprising, important aspects of nepotistic design appear to remain intact even when related emotional circuitry has been profoundly revised. Until such time as compelling evidence of dysfunction (rather than difference) is presented, it is imperative that we continue to develop and test alternative, functional hypotheses of psychopathy such as the frequency-dependent model posited by Harpending and Sobus (1987), Mealey (1995), Harris et al. (2001b), Lalumière et al. (2001), and others.

## Conflict of interest statement

The authors declare that the research was conducted in the absence of any commercial or financial relationships that could be construed as a potential conflict of interest.
